# Allelopatic Potential of *Dittrichia viscosa* (L.) W. Greuter Mediated by VOCs: A Physiological and Metabolomic Approach

**DOI:** 10.1371/journal.pone.0170161

**Published:** 2017-01-13

**Authors:** Fabrizio Araniti, Antonio Lupini, Francesco Sunseri, Maria Rosa Abenavoli

**Affiliations:** Dipartimento AGRARIA, Università Mediterranea di Reggio Calabria, – Località Feo di Vito, Reggio Calabria, Italy; University of Vigo, SPAIN

## Abstract

*Dittrichia viscosa (L*.*) W*. *Greuter* is a pioneer species belonging to the Compositae family. It is widespread in the Mediterranean basin, where it is considered invasive. It is a source of secondary metabolites, playing an important ecological role. *D*. *viscosa* plant extracts showed a phytotoxic activity on several physiological processes of different species. In the current study, the allelopathic potential of *D*. *viscosa* VOCs, released by its foliage, was evaluated on seed germination and root growth of lettuce. The VOCs effect was also studied on lettuce adult plants in microcosm systems, which better mimicked the open field conditions. *D*. *viscosa* VOCs inhibited both seed germination and root growth of lettuce. The VOCs composition revealed a large presence of terpenoids, responsible of the effects observed. Moreover, *D*. *viscosa* VOCs caused an alteration on plant water status accompanied by oxidative damages and photoinhibition on lettuce adult plants.

## Introduction

*Dittrichia viscosa (L*.*) W*. *Greuter (syn*. *Inula viscosa (L*.*) Aiton*., *Cupularia viscosa G*. *et G*.*)* is an evergreen perennial shrubby-weed belonging to the family Compositae. It is native to the Mediterranean region and it is considered a ruderal species due to its abundance in anthropic altered areas [1], and in particular, in metal-polluted sites [2]. *D*. *viscosa* has a remarkable pioneer nature since it colonizes different habitats in the Mediterranean basin where it often creates large monospecific communities [3]. In Australian and some European countries, this species is considered an important environmental weed for its high seed production and spreading, and for its resistance/adaptation to adverse conditions [3, 4]. *D*. *viscosa* is characterized by a quite substantial root apparatus and the ratio between below- and above-ground biomass is 0.24 [5]. The canopy is very dense reaching 150 cm of height and total leaf area per plant comprises 200 cm^2^ [6]. Glandular hairs, which produce a sticky resin from which derives “viscosa” name, cover the entire plant conferring its strong typical fragrance [3].

*D*. *viscosa* has allelopathic potential and, in particular, its extracts caused phytotoxic effects on several species, inhibiting roots and causing root anatomical abnormalities [7]. Interestingly, these extracts did not cause autotoxicity phenomenon conferring to this species a competitive advantage over other species [8]. Moreover, leaf epicuticular substances were also considered strong allelopatic agents for N_2_-fixing soil cyanobacteria, decreasing dramatically the photosynthetic assimilation of CO_2_ and increasing the heterocyst-to-vegetative cell ratio and most likely the assimilation of N_2_ of the cyanobacteria [9]. Finally, the effect of several extracts was assayed on crops and weeds, pointing out that *D*. *viscosa* allelopathic potential seems to be attributed mainly to leaf leachate [10]. This effect could be attributable to the presence of many secondary metabolites such as flavonoids [11], sesquiterpene lactones and acids [12–14] and triterpenoids [15, 16], biologically active compounds. In particular, formulated leaf extracts of *D*. *viscosa* exhibited nematicidal [17, 18], insecticidal [19, 20] antifungal [21, 22] activity.

Although many information is available regarding the *in-vitro* phytotoxic potential of the *D*. *viscosa* extracts, the activity of volatile organic compounds (VOCs), released by foliage, was never assessed. The presence of allelochemicals in aromatic shrub has been already established [23] playing a pivotal role especially in arid and semi-arid conditions where they act in the vapor phase [23, 24]. Several studies demonstrated that plant volatiles are potent seed germination inhibitors, reducing seedling establishment and growth in ecosystems [25–27]. For example, the VOCs produced by *Artemisia vulgaris* [26, 28], *Calamintha nepeta* or perennial groundcovers [29–32] suppressed weed seedling growth.

The VOCs are generally composed by terpenes, which provide to species a large number of ecological advantages [33] including plant reproduction, pollinator attractants, herbivores protection and plant–plant communication. These features make these compounds determinants for the vegetation patterning [34, 35].

In the current paper, the allelopathic potential of *D*. *viscosa* VOCs, naturally released from its foliage, was assessed on *Lactuca sativa* L. The VOCs effect was assayed on seed germinatin and root growth of lettuce. Furthermore, the response of lettuce adult plants to VOCs was also studied in microcosm systems, in order to better mimic the open field conditions. Furthermore, the metabolome profile of lettuce adult plants, in response to VOCs released by a donor species, was here analyzed for the first time.

## Materials and Methods

### Plant material

Aerial parts of *D*. *viscosa* were sampled on September 2015 in Calabria (Southern Italy). The plant material was collected in experimental fields belonging to the University Mediterranea of Reggio Calabria (Italy) (latitude N 38°7’ 40.883”, longitude E 15°40’ 36.375”) and its collection did not require any specific permission.

### *In vitro* VOCs bioassays

The *in vitro* volatiles bioassay was carried out as previously described by Araniti et al. [29], with some modifications. Freshly harvested greenish branches were evaluated for the bioactive volatile activity allowing only atmospheric contact between the test and the donor species. The aerial parts (0, 25, 50, 100 and 200 g of entire branches) of *D*. *viscosa* (donor species) were daily collected and immediately placed on the bottom of a 4.5 L jar capped with a plastic net (S1 Fig). A Petri dish (6 cm Ø) was filled with a double layer of filter paper, moistened with 2 ml of sterile deionized water and gently laid on a porous surface on the top of cut plant material (S1 Fig). The jar was then transferred in a ventilated climatic chamber settled with a temperature of 25±1°C and a 16:8 (light:dark) photoperiod. For each petri dish, 10 sterilized seeds of *L*. *sativa* L. (var. Parris Island COS), were sown. Germinated seeds were counted every 6 hours for 48 hr, considering germinated those seeds showing ≥ 1 mm of root protrusion from seed coat. According to Chiapusio et al. (1997), at the end of the observations, the total germination rate [G_T_ (%)] and the average speed of germination (S) were calculated as follow:

- Total germination rate:

$$G_{T}\;\left(\%\right)=100*(N_{T}/N)$$

where T indicates the last observation and N is the total number of seeds sown.

- Average speed of germination:

$$S=\left(N_{1}*1\right)+\;1/2\left(N_{2}-N_{1}\right)+1/3\;\left(N_{3}-N_{2}\right)+\ldots .+1/n\;(N_{n}-N_{n-1})$$

where N_1_, N_2_, N_3_, N_n-1_, N_n_ is the number of germinated seeds at each counting time.

Root growth [TRL (cm)] was evaluated on five pre-germinated seedlings, grown as previously described for seed germination, after 48 hr of treatment. *D*. *viscosa* plant material was daily changed to mimic field conditions.

#### Head space GC-MS analysis of plant volatiles

The *D*. *viscosa* VOCs were chemically characterized using a Thermo Fisher gas chromatograph apparatus (Trace 1310) equipped with a single quadrupole mass spectrometer (ISQ LT). The capillary column was a TG-5MS 30 m×0.25 mm×0.25μm the gas carrier was helium with a flow rate of 1 ml/min. Injector and source were settled at 200°C and 260°C, respectively. The sample (1 g of fresh plant material) was incubated for 1 minute at 40° and 1 μl of the head space was injected in split mode with a split ratio of 60. The following temperature was programmed: isocratic for 7 minutes at 45°C, from 45°C to 80°C with a rate of 10°C×min, from 80°C to 200°C with a rate of 20°C×min, then isocratic for 3 minutes 200°C. Mass spectra were recorded in electronic impact (EI) mode at 70 eV, scanning at 45–500 m/z range. Compounds identification was carried out comparing the relative retention time and mass spectra of molecules with those of the libraries (NIST 2005, Wiley 7.0 etc.).

### Bioassays on adult plants

#### VOCs bioassay on adult plants

The VOCs bioassay on lettuce adult plants was carried out as previously described [36]. Lettuce plants (30 days old) were exposed to *D*. *viscosa* VOCs, produced by 50 g of living plant material, for 12 days. During this period plants were daily irrigated with a half strength Hoagland solution. As for germination and root growth bioassays, *D*. *viscosa* plant material was daily renewed.

#### Photosynthetic pigments and Chlorophyll *a* fluorescence measurements

Chlorophyll a (Chl_a_), chlorophyll b (Chl_b_), and carotenoids (Ct) content were determined according to Wellburn [37]. In particular, 100 mg of frozen plant material were extracted with 1.5 ml of pure methanol and centrifuged at 170 *g* at 4°C for 5 min. Five hundred μL of supernatant were then collected and diluted with 500 μL of methanol. The absorbance of the pigment extract was determined at 470, 653, 666 and 750 nm. Pigment content was evaluated according to Wellburn’s equations [37].

The fluorescence of the chlorophyll *a* emitted by plants (three per treatment) exposed to VOCs was determined as described by Araniti et al. [36] with some modifications. Chlorophyll fluorescence was monitored using the Maxi-Imaging-PAM Chlorophyll Fluorescence System fluorometer (Walz, Effeltrich, Germany), and monitored every three days for 12 days. The following parameters were calculated: the maximum quantum efficiency of dark-adapted photosystem II (F_v_/F_m_); the maximum quantum efficiency of lighted photosystem II (*Φ*_II_); the regulated energy dissipation in the form of heat (*Φ*_NPQ_); the nonregulated energy dissipation (*Φ*_NO_, fluorescence emitted) and the estimated electron transport rate (ETR). The photosynthetic response was monitored for 5 min, and fifteen measurements were obtained for each parameter at each measuring time.

#### *In situ* semi-quantitative determination of H_2_O_2_

Hydrogen peroxide was determined according to Araniti et al. [38] with some modifications. Four fully expanded leaves were cut and fixed for 8 h in 3,3’-diaminobenzidine (DAB) (1 mg mL^-1^) solution (pH 3.8). The incubation was carried out in dark condition and pigments were successively removed rinsing leaves twice in pure ethanol. Bleached leaves were stored in 80% glycerol. Stained areas were determined by image analysis with the software Image ProPlus v.6.0 (Media Cybernetics Inc., Bethesda, MD, USA).

#### Lipid peroxidation

Lipid peroxidation was determined as described by Araniti et al. [36], measuring the increase of malondialdehyde (MDA) in the sample. Liquid nitrogen powdered plant material (100 mg) was homogenized in 80% cold ethanol solution (1 ml) and centrifuged at 3000 g for 10 minutes at 4°C. The supernatant was collected and incubated for 25 minutes at 95°C with 20% trichloroacetic acid (TCA) containing 0.01% hydroxytoluenebutylate, with and without 0.5% thiobarbituric acid (TBA). After incubation, the reaction was stopped in ice and the samples were centrifuged at 3000 g at 15°C for 10 minutes. The absorbance of the supernatant was measured at 440, 532 and 600 nm.

The equivalents of MDA were calculated using the equations proposed by Hodges et al. [39].

#### Total protein content

Total protein content of lettuce leaves exposed for 12 days to *D*. *viscosa* VOCs was determined according to Bradford [40], using bovine serum albumin as standard. Protein content was expressed as micrograms per gram of dry weight.

#### Fresh weight (FW), dry weight (DW), DW/FW ratio, leaf relative water content (RWC), leaf osmotic potential [Ψ (π)] and leaf membrane stability index [MSI (%)]

At the end of the experiment, four plants were collected and their FW was evaluated. Plants were oven dried for one week at 60°C in order to get DW and to calculate DW/FW ratio parameter.

Leaf relative water content (RWC) was estimated as previously reported by Mullan and Pietragalla [41] with some modifications. One leaf for each replicate was weighted (FW) and incubated for 24 h at 4°C in 50 ml falcon tube filled with 15 ml of ultrapure water. After incubation the turgid weight (TW) was taken and leaf samples were transferred in an oven and dried at 60°C for 48 h and successively weighted (DW). RWC parameter was evaluated using the following equation:

RWC=[(FW−DW)/(TW−DW)]*100

Leaf Ψπ was measured at the end of the experiment on four leaves per treatment (0 and 50 g cut plant material) as described by Araniti et al. [36]. Lettuce leaves were collected and frozen at -20°C. After 24 hours, leaves were squeezed into a syringe (the first drop was thrown away), the extract was collected and the Ψπ was measured with a cryoscopic osmometer (Osmomat 030, Gonatec).

The MSI was determined indirectly as reported by Saìram et al. [42] with some modifications. Leaf discs with uniform size were transferred in falcon tubes containing 10 ml of ultrapure water and heated at 30°C for 30 minutes. Then electrical conductivity was measured (C1) and the samples were immediately transferred at 100°C, for 30 minutes. Samples were cooled in ice and the electric conductivity was newly measured (C2). MSI index was obtained using the following equation:

$$SI\;\left(\%\right)=\left(1-\;C_{1}/C_{2}\right)*100$$

#### Extraction, identification and quantification of primary metabolites in lettuce leaves

Extraction, identification, and quantification of metabolites from lettuce leaves were performed as reported by Shi et al. [43], whereas sample derivatization were performed as Lisec et al. [44]. The derivatizated extract was injected into a TG-5MS capillary column using the single quadrupole gas chromatographer coupled to a mass spectrometer (GC-MS) (Thermo Fisher), as previously described.

Injector and source were settled at 250°C and 260°C temperature, respectively. One μl of sample was injected in splitless mode with a flow of 1ml/min using the following programmed temperature: isothermal 5 min at 70°C followed by a 5°C/ min ramp to 350°C and a final 5 min heating at 330°C. Mass spectra were recorded in electronic impact (EI) mode at 70 eV, scanning at 45–500 m/z range.

The extracted metabolites were identified comparing every retention time index-specific mass with reference spectra in mass spectral libraries (NIST 2005, Wiley 7.0 etc.). Relative metabolites quantification was based on a pre-added internal standard (ribitol at 0.02 mg/ml) added during the extraction process.

#### Experimental design and statistical analysis

A completely randomized design with four replications was applied in all the experiments except for chlorophyll *a* fluorescence that was replicated 3 times. Data were first checked for normality through the Kolmogorov-Smirnov test and then tested for homogeneity of variances with the Levene’s test. Differences among group means were statistically evaluated by analysis of variance followed by Least Significant Difference tests (LSD) in the case of homoscedastic data, and by Tamhane’s T2 test in the case of heteroscedastic data (*P* ≤ 0.05).

All statistical analyses were conducted using SPSS *ver*. 6.1 software (Insightful Corporation, USA). Speed Germination, Total Germination and Total Root Length responses to different doses of plant material were evaluated by a nonlinear regression model using a log-logistic function in order to calculate the ED_50_ values as reported by Araniti et al. [45–47].

Metabolite concentrations were checked for integrity and missing values were replaced with a small positive value (the half of the minimum positive number detected in the data). Data were successively normalized by a pooled sample (Creating a pooled average sample from control groups), transformed through “Log normalization” (to make the metabolite concentration values more comparable among different compounds) and scaled through Pareto-Scaling (mean-centered and divided by the square root of standard deviation of each variable) [48]. Data were then classified through Principal Component Analysis (PCA), metabolite variations were presented and samples clusterized through a heatmap. Differences between treatments were considered significant when the *P ≤* 0.05 (Student’s *t*-test).

Ingenuity pathway analysis was performed with MetPA, a web-based tool for pathway analysis & visualization metabolomics. MetPA is a module of Metaboanalyst that combines results from powerful pathway enrichment analysis with the pathway topology analysis to help researchers in identifying the most relevant pathways involved in the conditions under study. Compound ID associations were determined by matching to HMDB, PubChem and KEGG. Then, the pathway library, the algorithm for pathway enrichment analysis, and the algorithm for topological analysis were selected and performed to evaluate the possible biological impacts on the perturbed pathways [48].

## Results

### Experiments on lettuce seeds and seedlings

#### *In vitro* VOCs bioassay

**Fig 1:**
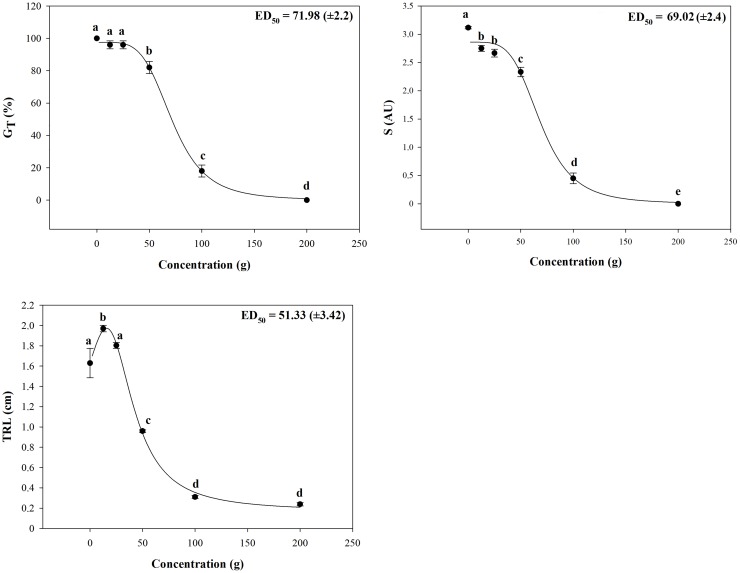
Effects of *Dittrichia viscosa* volatiles on germination and root growth. Effects of *D*. *viscosa* VOCs on Total Germination [G_T_ (%)], Speed Germination (S) and Total Root Length [TRL (cm)] of *L*. *sativa*. The nonlinear regression fitting of all the dose-response curves pointed out a significance level of *P* < 0.001. Different letters along the curve indicate statistical differences with *P* ≤ 0.05 (LSD). AU = arbitrary units. N = 4.

The results pointed out a strong phytotoxic potential of *D*. *viscosa* VOCs on both lettuce seeds germination and seedling growth. In particular, chemicals released from plant material strongly reduced, in a dose dependent manner, both speed (S) and total germination (G_T_). Interestingly, the S parameter was significantly inhibited (≈ 12%) even at the lower treatment (12.5 g), increasing up to 86% at 100 g of plant material (Fig 1). Conversely, the lowest treatments (12.5 and 25 g) did not affect the G_T_ parameter, while 50 g treatment caused 18% of inhibition, reaching the 82% at 100 g and 100% at the highest concentration (Fig 1). The nonlinear regression fit of both S and G_T_ raw data gave 69.02 g and 71.98 g ED_50_ values, respectively (Fig 1).

A stimulation of TRL in lettuce root seedlings, at the lowest concentration (12.5 g), was observed, whereas 25 g did not affect this parameter. On the contrary, higher doses strongly affected lettuce root growth causing 41%, 81% and 85% of inhibition at 50 g, 100 g and 200 g of plant material treatment, respectively. The non linear regression fit of TRL raw data pointed out 51.33 g ED_50_ value (Fig 1).

#### Head space GC-MS analysis of plant VOCs

The VOCs characterization released by *Dittrichia viscosa* leaves allowed the identification of 39 compounds, most of which belong to the terpenoids class (Table 1). In particular 4 aldehyde, 2 alcohols and 1 ester were identified (Table 1). Among terpenes, 16 monoterpenes (among them one monoterpenic ester and three monoterpenic alcohols), 1 homoterpene and 15 sesquiterpenes were identified (Table 1).

**Table 1:** Chemical characterization of *Dittrichia viscosa* VOCs. *Dittrichia viscosa* VOCs characterization. Aldehydes: 1–4; alcohols: 5–6; monoterpenes: 7–17, 19, 21–24; homoterpene: 20; sesquiterpenes: 25–39.

	^a^RT	Compound	Class	^b^RAP%
1	2.58	Isovaleraldehyde	Aldehyde	0.33
2	2.68	α-Methylbutanal	Aldehyde	0.56
3	5.53	n-Hexanal	Aldehyde	0.18
4	8	Leaf aldehyde	Aldehyde	0.39
5	8.26	3-Hexen-1-ol	Alcohol	0.52
6	8.82	3-Methylpentanol	Alcohol	0.17
7	10.2	α-Thujene	Monoterpene	3.26
8	10.37	α-Pinene	Monoterpene	9.88
9	10.78	Camphene	Monoterpene	0.3
10	11.38	Sabinene	Monoterpene	6.83
11	11.43	ß-Pinene	Monoterpene	3.37
12	11.74	Myrcene	Monoterpene	0.81
13	11.88	1,8-Cineole	Monoterpene	0.89
14	12.31	o-Cymene	Monoterpene	1.31
15	12.53	Eucalyptol	Monoterpene	43.24
16	12.82	γ-Terpinene	Monoterpene	1.47
17	13.05	cis-Sabinene hydrate	Monoterpene	0.93
18	13.36	Methyl benzoate	Ester	0.08
19	13.46	4-Terpinenyl acetate	Monoterpene esters	0.47
20	13.51	(3E)-4,8-Dimethyl-1,3,7-nonatriene	homoterpene	0.18
21	13.7	p-Menth-2-en-1-ol	Monoterpene alcohol	0.06
22	13.98	Camphor	Monoterpene	0.18
23	14.26	L-terpinen-4-ol	Monoterpene alcohol	0.3
24	14.4	α-Terpineol	Monoterpene alcohol	0.11
25	15.86	Ylangene	Sesquiterpene	1.84
26	15.9	α-Copaene	Sesquiterpene	1.36
27	15.94	α-Panasinsen	Sesquiterpene	0.44
28	16.03	Sativen	Sesquiterpene	0.12
29	16.24	(E)-Caryophyllene	Sesquiterpene	1.72
30	16.29	Isolongifolene	Sesquiterpene	0.14
31	16.37	Guaia-6,9-diene	Sesquiterpene	2.63
32	16.42	α-Gurjunene	Sesquiterpene	0.22
33	16.48	α-Humulene	Sesquiterpene	0.34
34	16.53	Alloaromadendrene	Sesquiterpene	0.22
35	16.62	α-Muurolene	Sesquiterpene	1.01
36	16.69	α-Cadinene	Sesquiterpene	2.99
37	16.76	α-Selinene	Sesquiterpene	0.2
38	16.9	Δ-Cadinene	Sesquiterpene	0.1
39	17.11	(E)-Nerolidol	Sesquiterpene	0.51

^a^ RT: retention time.

^b^ RAP% (Relative area percentage, peak area relative to total peak area %).

### Experiments on lettuce adult plants

#### In vivo Chlorophyll *a* fluorescence measurements and photosynthetic pigment content

**Fig 2:**
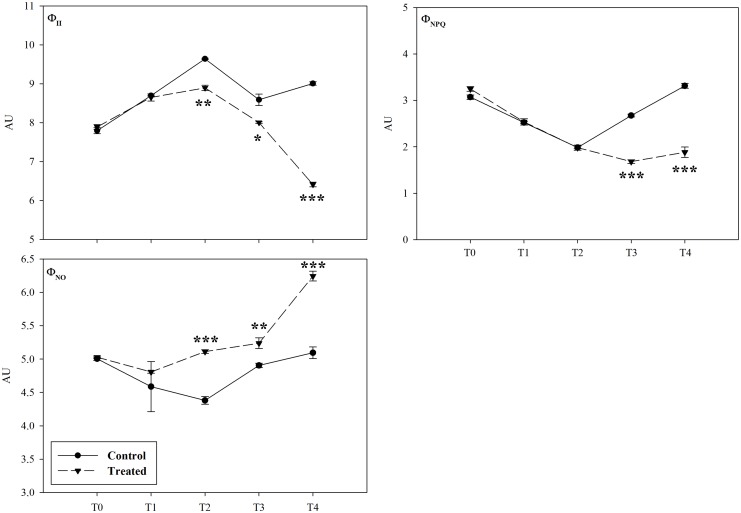
Effects of *Dittrichia viscosa* volatiles on photochemical quantum yield of the PSII, the quantum yield of light-induced nonphotochemical quenching and chlorophyll fluorescence. Values of the effective photochemical quantum yield of the light adapted PSII *Φ*_II_, the quantum yield of light-induced nonphotochemical quenching *Φ*_NPQ_ and chlorophyll fluorescence *Φ*_NO_ in whole lettuce plants after *D*. *viscosa* VOCs exposition (50 g of plant material). Asterisks along the curves indicate statistical differences with (*P* ≤ 0.05). * *p* < 0.05; ** *p* < 0.01; *** *p* < 0.001. T_0_ –T_4_ = days of treatment. AU = Arbitrary Units. N = 3.

**Fig 3:**
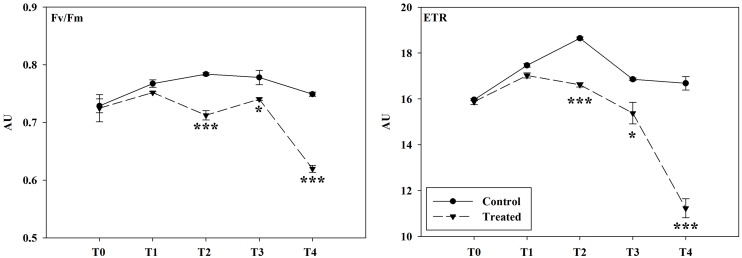
Effects of *Dittrichia viscosa* VOCs on maximum quantum efficiency of dark-adapted PSII and apparent electron transport rate. Values of maximum quantum efficiency of dark-adapted PSII (*Fv/Fm*) and apparent electron transport rate (*ETR*) in whole lettuce plants after *D*. *viscosa* VOCs exposition (50 g of plant material). Asterisks along the curves indicate statistical differences with (*P* ≤ 0.05). * *p* < 0.05; ** *p* < 0.01; *** *p* < 0.001. T_0_ –T_4_ = days of treatment. AU = Arbitrary Units. N = 3.

**Fig 4:**
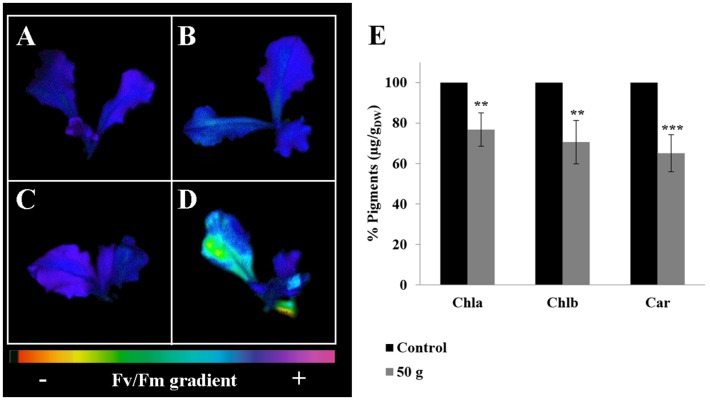
Pseudo-colour images of maximum quantum efficiency of dark-adapted PSII after the treatments of the plants with *Dittrichia viscosa* volatiles and their effects on pigments content. Pseudo-colour images of maximum quantum efficiency of dark-adapted PSII (Fv/Fm) in adult plants of lettuce after *D*. *viscosa* VOCs exposition. Images were taken at the beginning (T0) and at the end of the experiment (T4). **A**) Control plant at T0; **B**) control plant at T4; **C**) treated plant at T0; **D**) treated plant at T4. Images of the various fluorescence parameters are depicted in false colors coding from 0.0 (black) to 1.0 (purple). N = 3. **E)** Pigments content [μg/DW(g)]: Chlorophyll *a* (Chla), Chlorophyll *b* (Chlb), Carotenoids (Car). Data are given in percentage compared to the control and were analyzed trough LSD test. (*p* < 0.05). * *p* < 0.05; ** *p* < 0.01; *** *p* < 0.001. N = 3, for pseudo-color images; N = 4, for pigment content determination.

The monitoring of chlorophyll *a* fluorescence, emitted by lettuce adult plants exposed to *D*. *viscosa* VOC, pointed out strong inhibitory effects on photosynthetic activity (Figs 2–4). In particular, plants treated showed a high significant reduction on the photochemical quenching Φ_II_ from day 4 (T_2_) onwards (Fig 2), whereas Φ_NPQ_ parameter was significantly reduced only from day 6 (T_3_) until the end of the treatment (T_4_) (Fig 2). Conversely, the fluorescence parameter Φ_NO_ was significantly stimulated after 4 day (T_2_) of treatment (Fig 2). Finally, both F_v_/F_m_ and ETR parameters were significantly reduced after 4 days (T_2_) of treatment onwards (Fig 3). Interestingly, in the treated plants the F_v_/F_m_ clearly declined in the older peripheral leaves, which viability was irreversibly compromised, while the central vascular bundles and the youngest leaves were not affected (Fig 4A–4D).

Furthermore, 12 days of VOCs exposition caused a significant reduction of all the pigments content (Fig 4E). In particular, chlorophyll *a* and *b* were reduced by 23% and 30%, respectively. Interestingly, carotenoids were the most affected by VOCs exposition with 35% of reduction (Fig 4E).

#### FW, DW, DW/FW ratio, RWC, leaf Ψ (π), MSI (%), MDA (%), in situ semi-quantitative determination of H_2_O_2_ and protein quantification

**Fig 5:**
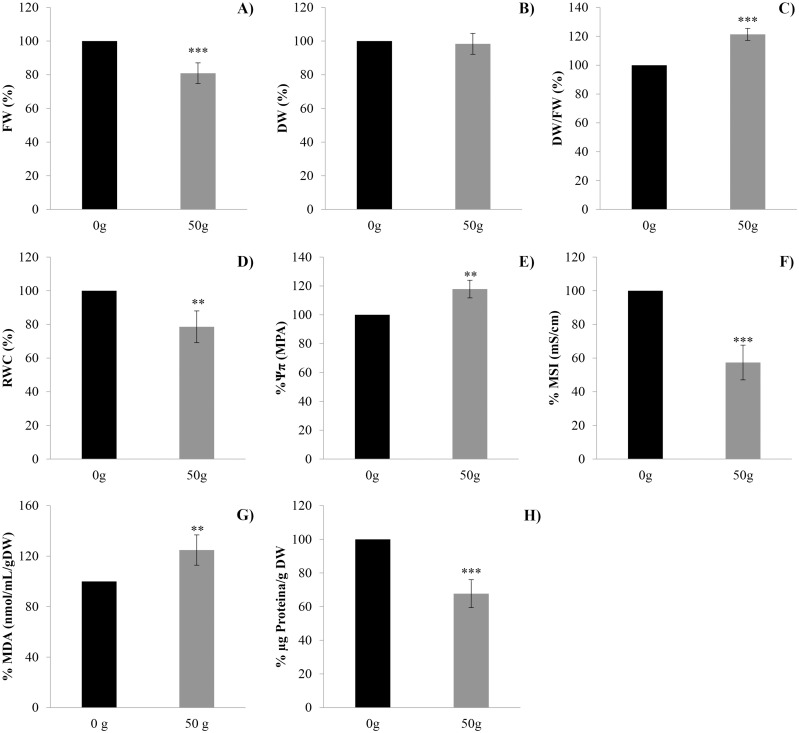
Effects of *Dittrichia viscosa* VOCs on several morphological and physiological parameters. Effects of *D*. *viscosa* VOCs on lettuce adult plants. A) Fresh weight (FW); B) dry weight (DW); C) DW/FW ratio; D) relative water content (RWC); E) leaf osmotic potential [Ψ(π)]; F) membrane stability index (MSI); G) lipid peroxidation (MDA) (nmol/mL/g_DW_); H) total protein content (μg of protein /g DW). Data are given in percentage compared to the control and were analyzed through LSD test. (*P* < 0.05). * *P* < 0.05; ** *P* < 0.01; *** *P* < 0.001. N = 4.

**Fig 6:**
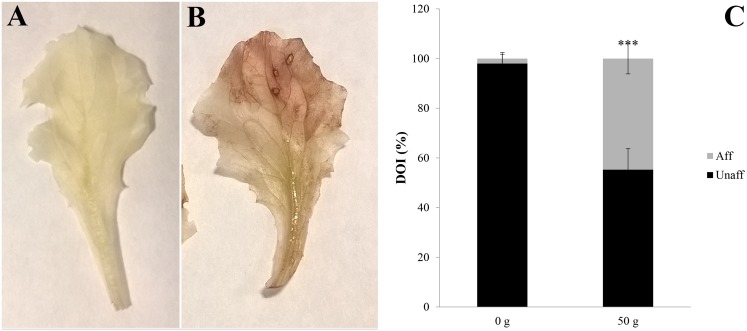
Semiquantitative determination of H_2_O_2_ in plants treated with *Dittrichia viscosa* volatiles. Lettuce leaves exposed to *D*. *viscosa* VOCs for 12 days showing the localization of the hydrogen peroxide on leaf surface after DAB staining: A) Control leaf; B) treated leaf; C) percentage of the Integrated Optical Density (IOD) obtained through image analysis carried on the *in situ* semi-quantitative determination of H_2_O_2_. In dark grey is reported the unaffected (Unaff) area of the leaf, whereas in bright grey the leaf surface interested by H_2_O_2_ accumulation (Aff). The area affected is expressed as percentage of the total area. (*P* < 0.05). * *P* < 0.05; ** *P* < 0.01; *** *P* < 0.001. N = 4.

Treatment of lettuce adult plants with *D*. *viscosa* VOCs (from 50 g plant material), for 12 days, caused 19% of FW reduction but did not significantly affect DW parameter (Fig 5A and 5B). Conversely, the DW/FW ratio significantly increased in treated plants (21%) (Fig 5C). Furthermore, the treated plants showed 22%, 32%, and 43% of reduction in RWC, protein content and MSI compared to control, respectively (Fig 5D, 5F and 5H). However, the Ψ(π) parameter was significantly stimulated (18%) (Fig 5E) as well as the lipid peroxidation (≈ 24%), indirectly determined by the increase of malondialdehyde (MDA) (Fig 5G). Finally, a high accumulation of H_2_O_2,_ which was present on almost the 44% of leaf surface, was observed (Fig 6).

#### Metabolomic experiments

To gain more insights into the modulation of metabolic homeostasis caused by *D*. *viscosa* VOCs exposition, GC-MS analysis were performed to identify differentially expressed metabolites. Fifty-four metabolites, including 10 amino acids, 23 organic acids, 9 sugars, 1 sugar acid, 5 sugar alcohols, 3 amines, 2 fatty acid and 1 glycan were examined in non-treated and treated plants.

**Fig 7:**
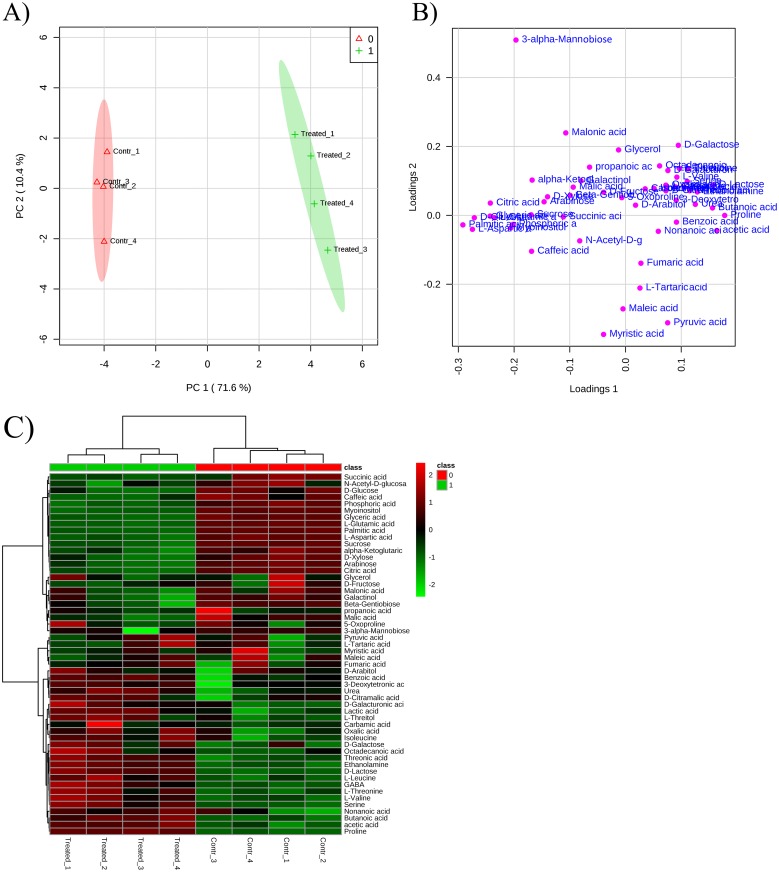
PCA analysis carried on the metabolite identified and quantified after *Dittrichia viscosa* VOCs treatment. **A)** Principal Component Analysis model scores **A)** and loading plot **B)** of metabolite profile of control plants (Contr_1 –Contr_4, replicates of control samples) and plants exposed to *D*. *viscosa* VOCs (Treated_1 –Treated_4, replicates of the treated samples). Both score and loading plots were generated using the first two PCs, PC1 *vs* PC2, with the explained variances shown in brackets; **C)** Overlay heat map of metabolite profiles in plants exposed to *D*. *viscosa* VOCs released by fresh aerial parts in comparison with control plants. Each square represents the effect of plant VOCs on the amount of every metabolite using a false-color scale. Red or green regions indicate increased or decreased metabolite content, respectively.

Metabolomic data were then analyzed through principal component analysis (PCA). In Fig 7A is reported the PCA score plot, which allowed samples separation and outliers detection basing on their metabolite profiles, whereas in Fig 7B is reported the PCA loading plot that allowed the identification of metabolites that contributes to the separation of samples reported on the score plot. This separation between control and treated samples was achieved using the principal components (PCs) PC1 *vs* PC2, which explained a total variance of 81.9%. In particular, PC1 explained the 71.6% of the variance while PC2 the 10.3%.

The PCA loading plot in Fig 7B shows that PC1 was dominated by D-Glucose, L-Aspartic acid and Palmitic acid, whereas PC2 was dominated largely by 3-α-Mannobiose.

PCA and heatmap visualization of metabolomic data showed distinct segregation between the control and the treated plants (Fig 7). Finally, the t-test analysis revealed 35 out of 55 significantly different metabolites between treated and non-treated samples (S1 Table).

**Fig 8:**
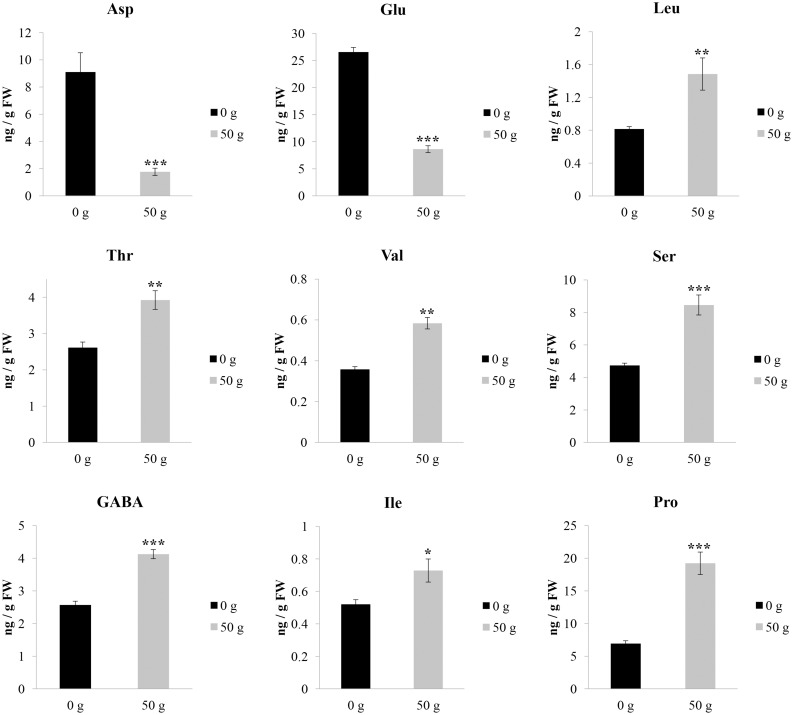
Effects of *Dittrichia viscosa* volatiles on amino acids content. The effects of the exposition for 12 days to *D*. *viscosa* VOCs on lettuce leaf amino acids abundance. Asp (aspartic acid); Glu (glutamic acid); Leu (leucine); Thr (threonine); Val (valine); Ser (serine); GABA (γ-aminobutiric acid); Ile (isoleucine); Pro (proline). Data were analyzed through t-test (P≤0.05) (data from S1 Table). * *P* < 0.05; ** *P* < 0.01; *** *P* < 0.001. N = 4.

**Fig 9:**
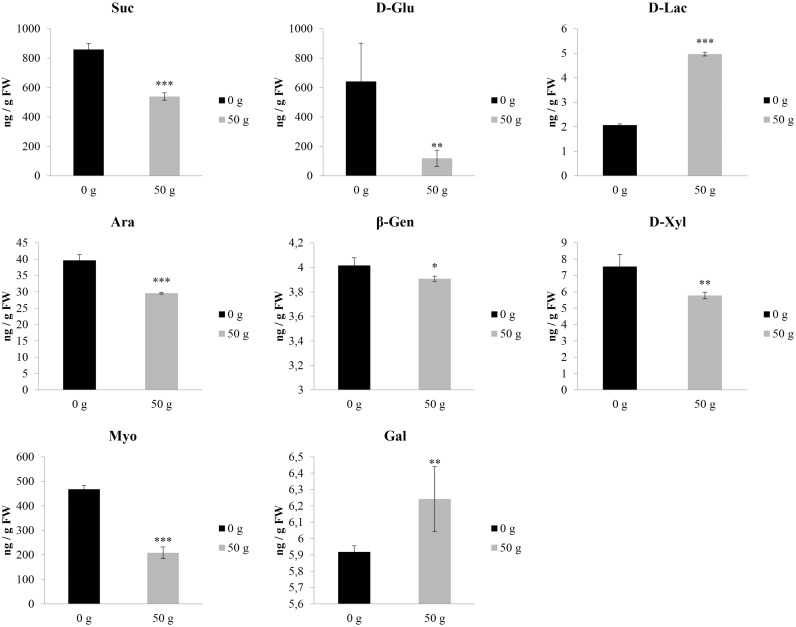
Effects of *Dittrichia viscosa* volatiles on sugars content. The effects of the exposition for 12 days to *D*. *viscosa* VOCs on lettuce leaf sugars abundance. Suc (Sucrose); D-Glu (D-Glucose); D-Lac (D-Lactose); Ara (Arabinose); β-Gentiobiose (β-Gen); D-Xyl (D-Xylose); Myo (Myoinositol); Gal (Galactinol). Data were analyzed through t-test (*P* ≤ 0.05) (data from S1 Table). * *P* < 0.05; ** *P* < 0.01; *** *P* < 0.001. N = 4.

A detailed analysis concerning the pathways and networks affected by the VOCs treatment was performed by MetPa. The “pathway analysis” of the results allowed to identify treatment impact on plant metabolism. Interestingly, several pathways were significantly affected (S2 Table), but the most effective and recurrent effects were mainly related to amino acids and sugars metabolism (S2 Table and Figs 8 and 9). In particular, L-aspartic (Asp) and L-glutamic (Glu) acids were reduced by 80.5% and 67.5%, respectively (Fig 8). Conversely, all the other amino acids were highly stimulated by VOCs exposition (Fig 8). In particular, L-leucine (Leu) and serine (Ser) content was ~ 80% higher than control, whereas an increase ranged between 40% and 63% of isoleucine (Ile), L-threonine (Thr), γ-aminobutiric acid (GABA) and L-valine (Val) content was observed. Finally, proline (Pro) was the most stimulated amino acids reaching values 1.8 folds higher than control (Fig 8). Among the sugars, sucrose (Suc) and glucose (D-Gu) content were reduced by ≈ 37% and 82%, respectively (Fig 9). Similarly, arabinose (25%), *β*-gentiobiose (3%), D-xylose (23%) and myoinositol (55%) where significantly reduced by the treatment (Fig 9), whereas D-lactose (140%) and Galactinol (5%) content was stimulated (Fig 9).

## Discussion

Consistent with previous results [9], the allelopathic potential of *D*. *viscosa* was confirmed through a bioassay that imitated natural environmental conditions of allelochemical VOCs release. For this reason, lettuce seeds, seedlings and adult plants were placed in open containers included in a ventilated growth chamber, and exposed to *D*. *viscosa* VOCs, directly released by plant parts. In particular, the effects of *D*. *viscosa* VOCs were demonstrated on lettuce seed germination and root growth, physiological processes largely employed to establish the secondary metabolites phytotoxicity [49] and the allelopathic potential of species [50].

*In vitro* results showed that *D*. *viscosa* VOCs had a strong inhibitory activity on both seed germination and root growth of lettuce resulting in low ED_50_ values. Similar effects were previously reported with *Calamintha nepeta* VOCs on germination and root growth of both crops and weeds [29], with *Salvia leucophylla* VOCs on root apical meristem of *Brassica campestris* [24] and with VOCs released by several selected ground covers on seeds [31]. Moreover, in agreement with several authors [51–53] the results clearly demonstrated that the seedlings establishment is more sensitive than germination to allelochemicals.

The effects of *D*. *viscosa* VOCs on seed germination were also analyzed comparing G_T_ (%) and S indexes. In particular, the G_T_ (%) parameter indicated that VOCs inhibited lettuce germination at relatively high concentrations. Conversely, the S index was significantly affected even at low concentrations. Juglone and *trans*-cinnamic acid caused similar effects on *A*. *thaliana* seeds [50]. The S reduction, which indicates a seed germination delay after VOCs exposure, is an important ecological effect because it represents a competitive advantageous strategy for *D*. *viscosa* in an ecosystem [54]. In fact, the germination delay could compromise, in the early stages of the seedlings life, the survival of the neighboring species. At this stage, a short period of inhibition could strongly reduce the ability of species to compete with others on their establishment [55, 56]. This could explain the ability of *D*. *viscosa* to create monospecific communities in its habitat.

Phytochemical analysis of *D*. *viscosa* VOCs revealed a strong presence of monoterpenes and sesquiterpenes, well known as allelopathic compounds, able to affect plant growth generally causing oxidative stress followed by a cascade effect on many physiological processes of receptor plants [57]. Both of them affected mitochondrial respiration [58, 59] and microtubule distribution and organization [38, 60], destroyed cell membranes [61] and altered hormone balance and plant water status [36, 62]. The sesquiterpenes *trans*-caryophyllene was able to affect the germination and growth of several weeds as well as of *Arabidopsis* adult plants inducing the alteration of plant water status [36]. Graña et al. [62] observed similar effects with the monoterpene citral. Singh et al. [63] reported that *α-*Pinene was able to inhibit root growth of several weeds causing oxidative stress and destroying cell membrane integrity. Moreover, Hussain et al. [64] demonstrated that the terpenoid artemisinin was able to inhibit photosynthetic efficiency, to cause oxidative stress and lipid peroxidation in roots as well as to interfere with calcium and nitrogen content in *Arabidopsis*.

Moreover, terpenoids, at non-phytotoxic concentrations, might act synergistically and become extremely phytotoxic [65], corroborating the hypothesis that allelopathic phenomenon is due to the combined action of different molecules, and underlining the complexity of plant interactions in natural- and agro-ecosystems.

Interestingly, the *D*. *viscosa* VOCs affected plant bio-membranes through the induction of lipid peroxidation resulting in a reduction of membrane stability (reduced MSI) and the semi-quantitative analysis of H_2_O_2_ further confirmed the presence of the oxidative damages. These effects were generally induced by allelochemicals, which inhibited the antioxidant enzymes activity increasing the level of free radicals, and consequently causing membrane lipid peroxidation and membrane potential alteration [66–70].

Under allelochemicals exposure, the oxidative stress was induced by plant water status alteration [36, 62]. For example, menthol and camphor, two terpenoids, enhanced the transpiration of *Arabidopsis thaliana* fully developed rosette dewaxing the lipophilic layers at leaf surface. As consequence, plants showed a dramatic effect characterized by water loss, necrosis and plant death, demonstrating that the lipophilic layers of leaf surface and stomata were primary targets of these monoterpenes [71]. Conversely, lettuce plants exposed to *D*. *viscosa* VOCs were extremely damaged without showing necrosis and/or plant death. This milder effect could be due to the different growth conditions, which ensured a greater air movement to plants avoiding VOCs accumulation.

Nevertheless, as suggested by Shultz et al. [71], high content and variability on terpenoids released by *D*. *viscosa* VOCs could affect leaf water status of lettuce, FW, DW/FW ratio, accompanied by the RWC reduction and the increase of Ψπ, typical of water status alteration.

The metabolomic profile of treated plants further confirmed this hypothesis, showing a high impact on amino acidic pathways, which results in an increment of several amino acids content, involved in osmotic adjustment. Among them, quaternary ammonium compounds [72–74] and amino acids such as proline, asparagine and γ-aminobutyric acid (GABA) [75–77] play a pivotal role in the recovery of plants from stress [77] such as osmotic stress [78–80]. In particular, proline accumulates to high levels in species adapted to arid and saline environments, in plant tissues withstanding severe desiccation, in response to allelochemicals [36, 62, 81, 82] and in cellular redox regulation, protecting proteins and membranes and scavenging reactive oxygen species [83, 84]. As well as proline, GABA, involved in many plant responses to stress, such as nitrogen storage, pH regulation, plant defense and osmotic adjustments [85], increased in treated plants. High increment of both proline and GABA could be correlated with the reduction of glutamic acid, a precursor of their synthesis [85, 86]. Indeed, previous experiments carried out with labeled (^14^C) gluthamic acid pointed out that water stressed shoots of bermuda grass readily accumulated much more proline newly synthesized from glutamic acid [87]. As well as glutamic acid, the aspartic acid content was also significantly reduced in plants exposed to *D*. *viscosa* VOCs, while a significant increment of its derivative threonine and isoleucine was observed. Previous studies demonstrated that aspartic acid and the derivative lysine, threonine and isoleucine represented building blocks for stress-specific proteins [88–90], allowing to justify their fluctuation in treated plants.

Interestingly, significant increase in leucine, valine concentration as well as in isoleucine, observed in treated plants, generally occurred under protein degradation, as in aged leaves before abscission or in plant cell resting cultures [91, 92].

Finally, oxidative stress, sucrose content reduction and strong increase of serine level in leaf suggested that plants are experiencing with the photorespiration rate increase as suggested by Bourguignon et al. [93]. In particular, serine was strongly involved in the photorespiratory cycle [93].

Plants exposed to *D*. *viscosa* VOCs reduced the levels of chlorophyll *a* and *b* as well as carotenoids, which are known to be accessory pigments with the important role as free radical scavengers protecting plants from photoinhibition [94]. The reduction of these pigments could enhance the ROS-mediated photodegradation of chlorophyll and the induction of photoinhibition [94, 95]. Therefore, plants, in order to protect the photosynthetic apparatus from photoinhibition, could adopt two strategies: first, the thermal dissipation of the energy in excess in the PSII antennae (nonphotochemical quenching), and second, the ability of PSII to transfer electrons to various acceptors within the chloroplast (photochemical quenching) [96]. If plants are not able to apply at least one of the two strategies, photoinhibition occurs. The monitoring of some photosynthetic parameters supported this hypothesis. In fact, plants exposed to *D*. *viscosa* VOCs reduced the light adapted photosystem II efficiency (Φ_II_) accompanied by an increase in the emission of non-regulated energy dissipation (Φ_NO_) and a reduction of the ETR. Moreover, treated plants showed a strong reduction in nonphotochemical quenching (Φ_NPQ_), which suggested that plants were not able to dissipate the energy in heat form [97]. Finally, the progressive reduction of the parameter Fv/Fm suggested a physical damage to the antenna complex, probably due to the high presence of ROS and lipid peroxidation.

Taken together these results suggested that both photochemical and biosynthetic phases of photosynthesis were directly affected by VOCs [97] and the confirmation was given by the sharp alteration of sugars metabolism and, in particular, by the reduction in sucrose and glucose content observed after treatment. In particular, the reduction in glucose, sucrose content was already observed in Arabidopsis cells exposed to oxidative stress [98] and in *Aegilops geniculate* plants treated with ferulic and *p*-coumaric acids [99], largely known for affecting photosynthesis [100].

## Conclusions

The results gave the first evidence of the allelopathic potential of *D*. *viscosa* through a bioassay that mimed natural environmental conditions of VOCs release. The effects of VOCs on plant-plant interaction and communication were studied through a physiological and metabolomic approach, for the first time.

*D*. *viscosa* VOCs have a strong inhibitory activity on both germination and root growth of *L*. *sativa* as well as on lettuce adult plants, where strongly affected their metabolism. High production of ROS accompanied by lipid peroxidation, membrane leakage and water status alteration were observed. Interestingly, plants tried to cope with this stress activating metabolic pathways involved in osmotic adjustment and radical scavenging activity. Nevertheless, lettuce plants exposed to VOCs suffered severe damages in the photosynthetic apparatus. In particular, damage to the antenna complex, pigment degradation, reduction of light adapted photosystem II efficiency, reduction of both electron transport rate and nonphotochemical quenching and increment in chlorophyll *a* fluorescence were caused by VOCs exposure on lettuce plants.

Taken together, these results suggested that plants exposed to *D*. *viscosa* VOCs were subjected to a cascade of events involving oxidative stress, photosynthesis machinery and process, water status, to which plant tried to cope by activating defense mechanisms including change in amino acidic metabolism.

In conclusion, *D*. *viscosa* is an allelopathic species that can induce net changes in its natural environment affecting nearby plants, and thus contribute to define plant community in the longer term.

## Supporting Information

S1 Fig Scheme of the in-vitro bioassay. Schematic representation of the experiments carried on seeds, seedlings and adult plants of lettuce.
(TIF)

S1 Table Quantification and statistical significance of the metabolites identified in control and treated plants. Chemical compounds isolated and quantified through GC-MS and significantly affected by the exposition for 12 days to *D*. *viscosa* volatiles. Data are expressed in nanograms/100mg of fresh plant material.
(DOCX)

S2 Table Impact of *Dittrichia viscosa* volatiles on plant metabolism. Result from “*Pathway Analysis*” carried on the concentrations of metabolite identified in lettuce plants treated for 12 days with *D*. *viscosa* VOC.
(DOCX)
