# Supplementary figures and images for: Allelopatic Potential of Dittrichia viscosa (L.) W. Greuter Mediated by VOCs: A Physiological and Metabolomic Approach
Source: PLoS One. 2017 Jan 13;12(1):e0170161. doi: 10.1371/journal.pone.0170161 (PMC5234817; doi:10.1371/journal.pone.0170161)

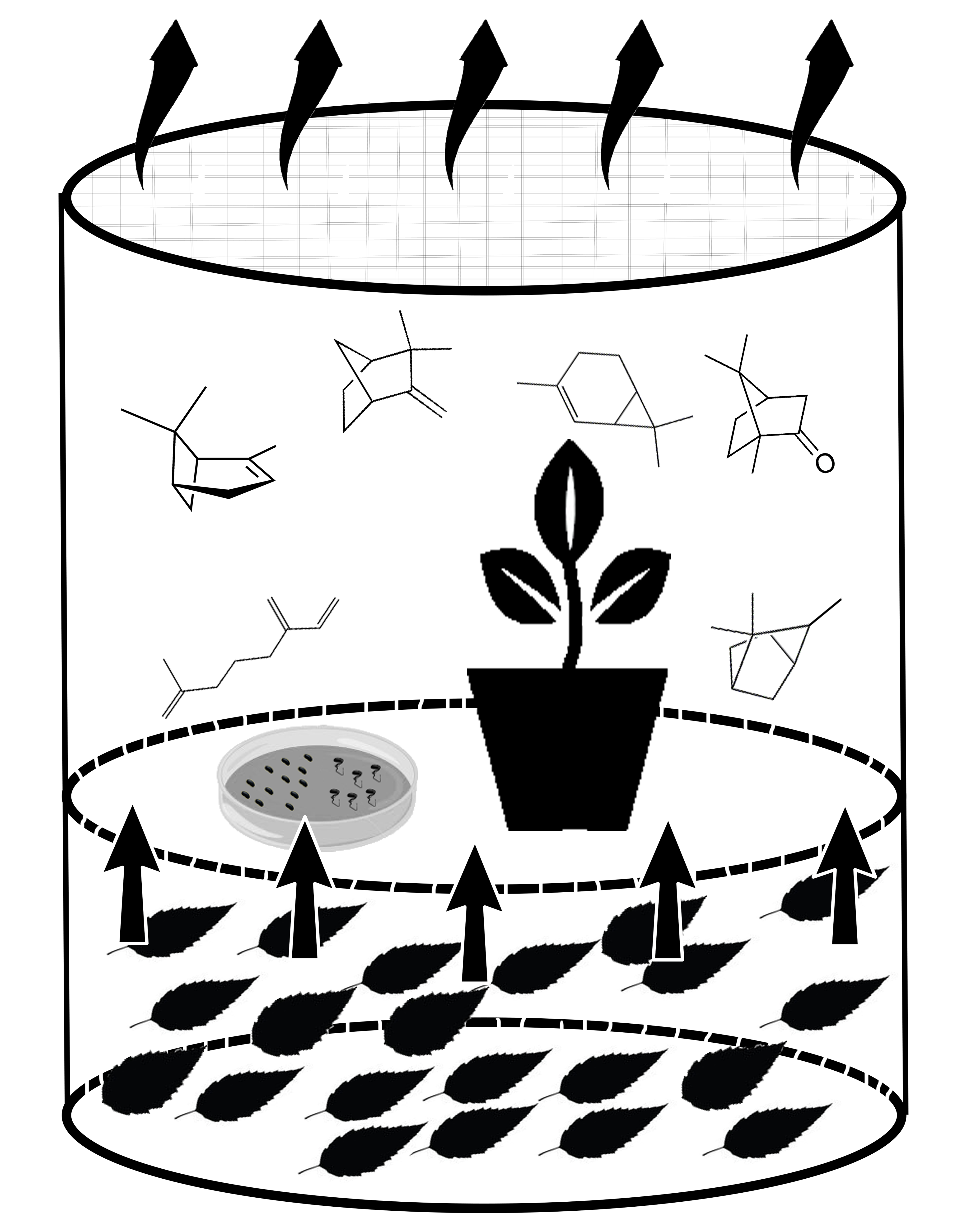

Supplement: S1 Fig — Schematic representation of the experiments carried on seeds, seedlings and adult plants of lettuce. (TIF) [file pone.0170161.s001.tif]
