# Supplementary material for: Allelopatic Potential of Dittrichia viscosa (L.) W. Greuter Mediated by VOCs: A Physiological and Metabolomic Approach
Source: PLoS One. 2017 Jan 13;12(1):e0170161. doi: 10.1371/journal.pone.0170161 (PMC5234817; doi:10.1371/journal.pone.0170161)
Supplement: S1 Table — Chemical compounds isolated and quantified through GC-MS and significantly affected by the exposition for 12 days to D. viscosa volatiles. Data are expressed in nanograms/100mg of fresh plant material. (DOCX) [file pone.0170161.s002.docx]

S1 Table. **Quantification and statistical significance of the metabolites identified in control and treated plants.** Chemical compounds isolated and quantified through GC-MS and significantly affected by the exposition for 12 days to *D. viscosa* volatiles. Data are expressed in nanograms/100mg of fresh plant material.

| **Feature** | **Control** | **Treated** | ***P* value** | **Class** |
| --- | --- | --- | --- | --- |
| 5-Oxoproline | 7.8928876 | 9.9617602 | NS | **Amino acid** |
| GABA | 2.5694369 | 4.1257531 | 0.0018568 |  |
| Isoleucine | 0.5208727 | 0.7285062 | 0.04372 |  |
| L-Aspartic acid | 9.1063652 | 1.7713658 | 2.14E-06 |  |
| L-Glutamic acid | 26.564405 | 8.6257049 | 1.23E-08 |  |
| L-Leucine | 0.814999 | 1.4850778 | 0.0025279 |  |
| L-Threonine | 2.6153406 | 3.9220986 | 0.0035248 |  |
| L-Valine | 0.3581496 | 0.5838498 | 0.0022189 |  |
| Proline | 6.9335823 | 19.228313 | 1.06E-07 |  |
| Serine | 4.7430399 | 8.4589592 | 0.0003367 |  |
| 3-Deoxytetronic acid | 0.6326889 | 1.0634423 | NS | **Organic acid** |
| Acetic acid | 0.4483552 | 1.133519 | 0.0001438 |  |
| α-Ketoglutaric acid | 2.656385 | 1.6238323 | 0.0006744 |  |
| Benzoic acid | 0.7100827 | 1.1568488 | 0.02851 |  |
| Butanoic acid | 0.1608758 | 0.3788809 | 9.93E-05 |  |
| Caffeic acid | 65.368257 | 38.081137 | 0.002262 |  |
| Carbamic acid | 7.3326832 | 9.9411699 | NS |  |
| Citric acid | 43.727711 | 13.095441 | 2.48E-06 |  |
| D-Citramalic acid | 0.7709962 | 1.2005053 | NS |  |
| Fumaric acid | 4.7484285 | 6.3572298 | NS |  |
| Lactic acid | 26.267984 | 46.134659 | 0.0074007 |  |
| L-Tartaric acid | 0.9719068 | 1.3302781 | NS |  |
| Maleic acid | 13.484363 | 16.338347 | NS |  |
| Malic acid | 544.44937 | 538.86275 | 0.010017 |  |
| Malonic acid | 1.9373568 | 1.6464874 | NS |  |
| Nonanoic acid | 1.4173144 | 2.0598935 | NS |  |
| Octadecanoic acid | 12.352673 | 18.216975 | 0.045247 |  |
| Oxalic acid | 97.877766 | 150.96112 | NS |  |
| Phosphoric acid | 34.749473 | 15.613555 | 2.26E-05 |  |
| propanoic acid | 0.5565723 | 0.4915729 | NS |  |
| Pyruvic acid | 0.6248993 | 1.084486 | NS |  |
| Succinic acid | 13.409082 | 11.424588 | 0.012223 |  |
| Threonic acid | 5.2739987 | 9.4972388 | 0.0001679 |  |
| Mannobiose | 2.1963658 | 1.2692456 | NS | **Sugar** |
| ß-Gentiobiose | 4.0151985 | 3.9072166 | 0.0042004 |  |
| D-Fructose | 1931.1699 | 2255.302 | NS |  |
| D-Galactose | 52.958587 | 94.075897 | NS |  |
| D-Glucose | 641.94738 | 119.15943 | 0.0017106 |  |
| Lactose | 2.0710543 | 4.9676715 | 6.54E-06 |  |
| Arabinose | 39.640959 | 29.595761 | 3.13E-05 |  |
| D-Xylose | 7.5494055 | 5.7749264 | 0.000477 |  |
| Sucrose | 859.42105 | 539.15808 | 1.35E-06 |  |
| Glyceric acid | 290.05118 | 86.579401 | 6.90E-08 | **Sugar acid** |
| D-Arabitol | 6.6578221 | 8.6972591 | NS | **Sugar alcohol** |
| Galactinol | 5.9182019 | 6.2416585 | 0.015055 |  |
| Glycerol | 7.4901963 | 9.2861453 | NS |  |
| L-Threitol | 1.1003043 | 1.9882404 | NS |  |
| Myoinositol | 467.84029 | 208.63518 | 1.76E-08 |  |
| Urea | 2.7488447 | 5.4102036 | 0.012898 | **Amine** |
| Ethanolamine | 1.4054865 | 2.7423699 | 3.06E-05 |  |
| N-Acetyl-D-glucosamine | 2.7987943 | 2.8215482 | 0.02859 |  |
| Myristic acid | 3.0688312 | 2.8318027 | NS | **Fatty acid** |
| Palmitic acid | 24.985237 | 3.9107881 | 8.12E-09 |  |
| D-Galacturonic acid | 2.298801 | 3.5685759 | 0.017746 | **Glycan** |

Important features selected by *t*-tests with threshold *P* ≤ 0.05. N=4. NS: Not Significant features.
