# Supplementary material for: Allelopatic Potential of Dittrichia viscosa (L.) W. Greuter Mediated by VOCs: A Physiological and Metabolomic Approach
Source: PLoS One. 2017 Jan 13;12(1):e0170161. doi: 10.1371/journal.pone.0170161 (PMC5234817; doi:10.1371/journal.pone.0170161)
Supplement: S2 Table — Result from “Pathway Analysis” carried on the concentrations of metabolite identified in lettuce plants treated for 12 days with D. viscosa VOC. (DOCX) [file pone.0170161.s003.docx]

S2 Table. **Impact of *Dittrichia viscosa* volatiles on plant metabolism.** Result from “*Pathway Analysis*” carried on the concentrations of metabolite identified in lettuce plants treated for 12 days with *D. viscosa* VOC.

| **Pathways** | **Total Cmpd** | **Hits** | ***P* value** | **Impact** |
| --- | --- | --- | --- | --- |
| Alanine, aspartate and glutamate metabolism | 22 | 7 | 7.67E-06 | 0.63218 |
| Galactose metabolism | 26 | 7 | 2.1004E-05 | 0.50774 |
| Glycine, serine and threonine metabolism | 30 | 4 | 5.60E-06 | 0.31657 |
| Inositol phosphate metabolism | 24 | 1 | 1.76E-08 | 0.25131 |
| Citrate cycle (TCA cycle) | 20 | 5 | 0.000392 | 0.2478 |
| Arginine and proline metabolism | 38 | 6 | 3.20E-09 | 0.2437 |
| Pyruvate metabolism | 21 | 3 | 0.002362 | 0.20409 |
| Methane metabolism | 11 | 1 | 0.000337 | 0.16667 |
| Sulfur metabolism | 12 | 2 | 3.14E-05 | 0.13333 |
| Glycolysis or Gluconeogenesis | 25 | 3 | 0.002362 | 0.11063 |
| Glyoxylate and dicarboxylate metabolism | 17 | 2 | 2.31E-05 | 0.10544 |
| Aminoacyl-tRNA biosynthesis | 67 | 7 | 1.03E-08 | 0.09302 |
| Starch and sucrose metabolism | 30 | 4 | 0.000146 | 0.08938 |
| Glutathione metabolism | 26 | 2 | 1.18E-08 | 0.07756 |
| Carbon fixation in photosynthetic organisms | 21 | 2 | 3.40E-05 | 0.0331 |
| Valine leucine and isoleucine biosynthesis | 26 | 5 | 0.000475 | 0.01865 |
| Purine metabolism | 61 | 1 | 0.012898 | 0.01071 |
| Selenoamino acid metabolism | 19 | 1 | 0.000144 | 0.00254 |

Total Cmpd: the total number of compounds in the pathway; Hits: is the actually matched number from the uploaded data; *P* value: is the original *P* value calculated from the enrichment analysis; Impact: is the pathway impact value calculated from pathway topology analysis.
